# Kernel dynamic orthonormal subspace analysis for monitoring hybrid electric vehicle powertrain faults

**DOI:** 10.1038/s41598-026-53315-8

**Published:** 2026-05-21

**Authors:** Yonghui Wang, Xiongshi Wang, Bian Gong, Syamsunur Deprizon, Heyan Li

**Affiliations:** 1 College of Urban Transportation and Logistics, Shenzhen Technology University, Shenzhen, China; 2 Faculty of Engineering, Technology and Built Environment, UCSI University, Kuala Lumpur, Malaysia

**Keywords:** Energy science and technology, Engineering, Mathematics and computing

## Abstract

Hybrid electric vehicle (HEV) powertrain systems exhibit complex dynamic and nonlinear characteristics due to the coupling effects among mechanical, electrical, and thermal subsystems. Traditional multivariate statistical process monitoring (MSPM) methods based on the time lag shift method (TLSM) may suffer from redundant information problems where historical data not used for prediction can contaminate the extracted features and reduce fault detection sensitivity. To address this limitation, this paper proposes a kernel dynamic orthonormal subspace analysis (KDOSA) method for monitoring HEV powertrain faults. The proposed method extends the OSA framework to the kernel feature space using Gaussian kernel functions, aiming to capture nonlinear dependencies while maintaining orthogonal separation between dynamic and static components. By decomposing real-time data into dynamic and static subspaces in the reproducing kernel Hilbert space, KDOSA is designed to mitigate the redundant information problem inherent in TLSM-based kernel methods such as dynamic kernel PCA. A comprehensive monitoring framework is developed with $T^2$ indices for both dynamic and static subspaces, providing fault detection capability and diagnostic information about fault origins. The effectiveness of the proposed method is examined through numerical simulations and real-world HEV powertrain experiments. Experimental results demonstrate that KDOSA achieves favorable fault detection performance with performance index (PI) values exceeding 95% across all test scenarios without triggering false alarms in the tested cases, showing improved performance compared with existing OSA-based nonlinear dynamic methods.

## Introduction

**Fig. 1:**
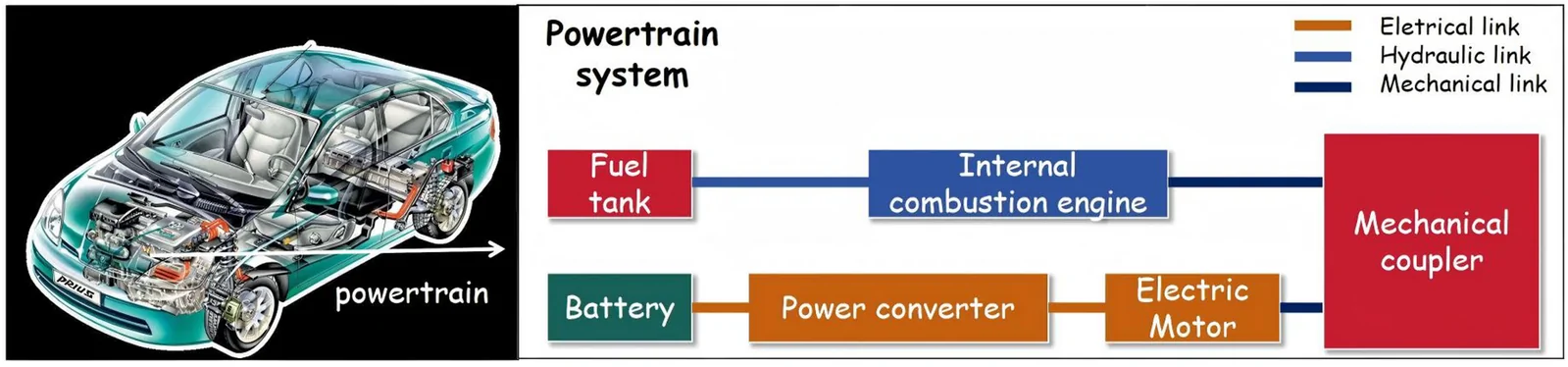
Structure of HEV powertrain system.

With the rapid development of hybrid electric vehicles (HEVs), the complexity of powertrain systems has increased significantly. As shown in Fig. 1, modern HEV powertrain systems consist of multiple interconnected components, including internal combustion engines, electric motors, battery packs, and power converters, all of which operate under highly dynamic and nonlinear conditions^1,2^. In light of safety concerns, faults that occur in powertrain systems can be crucial since an unexpected torque can risk the powertrain performance and consequently endanger passenger safety. Therefore, ensuring the safety, reliability, and efficiency of such systems is critical, as even minor faults can lead to performance degradation, energy loss, or system failure^3,4^.

To address these challenges, powertrain health management has emerged as a systematic framework that encompasses the monitoring, diagnosis, prognosis, and maintenance of complex systems throughout their operational lifecycle. Currently, the prevalent health management methods for powertrain systems include:

- Manual experience-based method: Relies on expert knowledge to assess vehicle condition through visual inspection, sound monitoring, and tactile examination without full disassembly. However, this method is slow, subjective, and incapable of quantitative analysis^5^.
- Testing and diagnostic method: Employs specialized instruments to collect operational parameters, curves, and waveforms for quantitative condition assessment without disassembly. Despite offering high accuracy and speed, it requires costly equipment and specially trained operators^5^.
- Self-diagnostic method: Utilizes onboard computers to automatically monitor system operation and record diagnostic trouble codes. While enabling automated fault logging, the resulting codes still require expert interpretation with technical documentation to identify root causes^6^.

An inherent limitation of the methods mentioned above lies in their reliance on expert experience, which hinders their adaptability for effective HEV powertrain system fault detection. In contrast, data-driven methods require little priori knowledge of powertrain system configurations and integrations and only rely on previously observed vehicle data^7,8^.

Data-driven methods for health management can be broadly categorized into two complementary directions. The first direction focuses on remaining useful life (RUL) prediction, which aims to estimate the degradation trajectory and forecast the time remaining before system failure. To address the scarcity of labeled degradation data, Zhang et al. proposed a self-attention mechanism-sequential variational autoencoder (SAM-SVAE) that jointly exploits labeled and unlabeled samples for RUL prediction with uncertainty quantification^9^. For cross-domain scenarios where data privacy must be preserved, Zhang et al. further developed a source-free domain adaptation framework based on DAGCN within distributed federated learning (DAGCN-SFDA-DFL), enabling knowledge transfer across facilities without sharing raw data^10^. Despite these advances, RUL prediction focuses on estimating when a system will fail rather than detecting whether an anomaly has already occurred in the current process state.

The second direction, multivariate statistical process monitoring (MSPM)^11–13^, addresses this complementary challenge by detecting faults in real time, which is particularly critical for HEV powertrains where timely anomaly identification is essential for preventing sudden failures. The MSPM methods project high-dimensional data into several low-dimensional subspaces, enabling the monitoring of large-scale processes. The most commonly used MSPM methods are principal component analysis (PCA)^14,15^ and independent component analysis (ICA)^16^, which analyze the correlation between variables and extract low-dimensional principal components (PCs) for fault detection. For traditional MSPM methods, PCs can be regarded as linear combinations of original process variables, which are not concerned with the autocorrelation relationship among the process variables.

For most industrial processes, especially HEV powertrains, the current state is also affected by the past state, that is, the process has a dynamic property, which is a challenge for traditional MSPM methods. The time lag shift method (TLSM)^17–19^ is the most common approach to the dynamic issue, whose idea is to add the historical data as additional variables to the original data matrix and monitor them with traditional MSPM methods. As such, MSPM methods can simultaneously analyze the static cross-correlation and dynamic autocorrelation among the process variables. As a simple and effective method, TLSM has been transplanted into most mainstream MSPM methods. TLSM was first applied in PCA by Ku et al. in 1995 as dynamic PCA (DPCA)^20^, and Lee et al. proposed dynamic independent component analysis (DICA)^21^ in 2004.

In addition to the dynamic characteristics, HEV powertrain systems also exhibit significant nonlinear behavior due to the complex coupling effects among mechanical, electrical, and thermal subsystems. To address this limitation, kernel methods have been integrated with TLSM-based approaches. Kernel methods map the original data into a high-dimensional feature space where nonlinear relationships become linear, enabling the application of linear techniques^22–24^. Notable examples include dynamic kernel PCA (DKPCA)^25^ and dynamic kernel ICA (DKICA)^26^, which have demonstrated improved performance for systems with dynamic and nonlinear characteristics.

It is worth noting that TLSM-based dynamic approaches incorporate historical data as additional real-time variables, which introduces a subspace division problem. Specifically, redundant historical information contaminates the extracted features, and predictable information is double-counted across real-time and historical data, leading to biased PC selection and reduced fault detection sensitivity. A rigorous mathematical analysis of this problem is provided in “Drawbacks of TLSM-based dynamic approaches”.

Orthonormal subspace analysis (OSA)^27–29^ was first proposed to handle the key performance indicator (KPI) monitoring issue. It can divide the process and KPI data into three orthonormal subspaces, containing KPI-related components, KPI-unrelated components in the input data, and input-unrelated components in the output data. For OSA, all subspaces are orthonormal, so OSA is also an effective subspace division method. To address the subspace division problem in TLSM-based dynamic approaches, Lou et al. adopted OSA^30^ to handle dynamic features, by taking real-time data as the output of OSA and historical data as input. OSA can successfully separate the real-time data into two groups: information that can be predicted by historical data (the dynamic component), and information that cannot be predicted for process monitoring (the static component). As such, the variance of the information that can be predicted by historical data is not counted repeatedly, and information not used for prediction does not participate in the selection of PCs.

However, the OSA-based dynamic method proposed by Lou et al. assumes linear relationships among variables, which limits its effectiveness in monitoring HEV powertrain systems that exhibit significant nonlinear behavior. Although kernel-based dynamic methods such as DKPCA and DKICA can handle nonlinear relationships, they still inherit the redundant information problem from the TLSM-based augmentation strategy. Therefore, extending the OSA-based dynamic method to handle nonlinear processes constitutes highly meaningful research. In this paper, we propose a kernel dynamic OSA (KDOSA) method that addresses the nonlinearity limitation while preserving the advantage of eliminating redundant information. The main contributions of this paper are summarized as follows:

1. Kernel extension for nonlinear dynamic monitoring: we extend the OSA-based dynamic method with kernel methods to handle nonlinear relationships in HEV powertrain systems. By employing Gaussian kernel functions to map the data into a reproducing kernel Hilbert space (RKHS), the proposed KDOSA can effectively capture complex nonlinear dependencies while preserving the orthogonal subspace structure for dynamic-static separation.
2. Theoretical analysis of redundancy elimination in kernel space: we provide a rigorous theoretical analysis demonstrating that the proposed KDOSA method successfully eliminates the redundant information problem in the kernel feature space. Unlike TLSM-based kernel methods such as DKPCA, which inherit the redundancy problem, KDOSA ensures that irrelevant historical information does not contaminate the extracted features, thereby improving fault detection sensitivity.
3. Theoretical analysis of fault severity sensitivity: we establish theoretical bounds for minimum detectable fault severity in both dynamic and static subspaces. This analysis proves that KDOSA maintains consistent sensitivity across varying fault magnitudes. This theoretical guarantee is absent in existing kernel-based and dynamic methods. The experimental results validate the theoretical analysis, showing that the proposed method achieves high detection sensitivity once the fault severity exceeds the minimum detectable threshold.
4. Comprehensive monitoring framework: based on the orthogonal decomposition in kernel space, we design a comprehensive monitoring framework that includes $T^2$ indices for both dynamic and static subspaces. This framework enables effective fault detection and provides diagnostic information about whether faults originate from dynamic or static process variations.

Finally, the proposed method is validated through both numerical simulations and real-world HEV powertrain data experiments, demonstrating significant improvements over existing methods in detecting faults under dynamic and nonlinear operating conditions.

## Preliminaries and problem statement

### TLSM-based dynamic approaches

Take ${{\bf X}}\in \mathbb {R}^{n\times m}$ (where *n* is the number of samples and *m* is the number of variables) as the process data. To handle dynamic features in process data, TLSM-based dynamic approaches construct a new data matrix as follows:

1 $$\begin{aligned} \tilde{{{\bf X}}} = \left[ {{\bf X}}(0), {{\bf X}}(-1), \cdots , {{\bf X}}(-q) \right] , \end{aligned}$$

where ${{\bf X}}(-j)$ is the variable shifted *j* times into the past (i.e., with *j* lags), and *q* is the maximum time lag. The new data matrix $\tilde{{{\bf X}}}$ is monitored by traditional MSPM methods such as PCA, ICA, and CVA. Based on Eq. (1), we see that $\tilde{{{\bf X}}}$ contains both autocorrelation and cross-correlation characteristics, so the corresponding TLSM-based dynamic approaches can simultaneously handle static and dynamic relationships.

### OSA

**Fig. 2:**
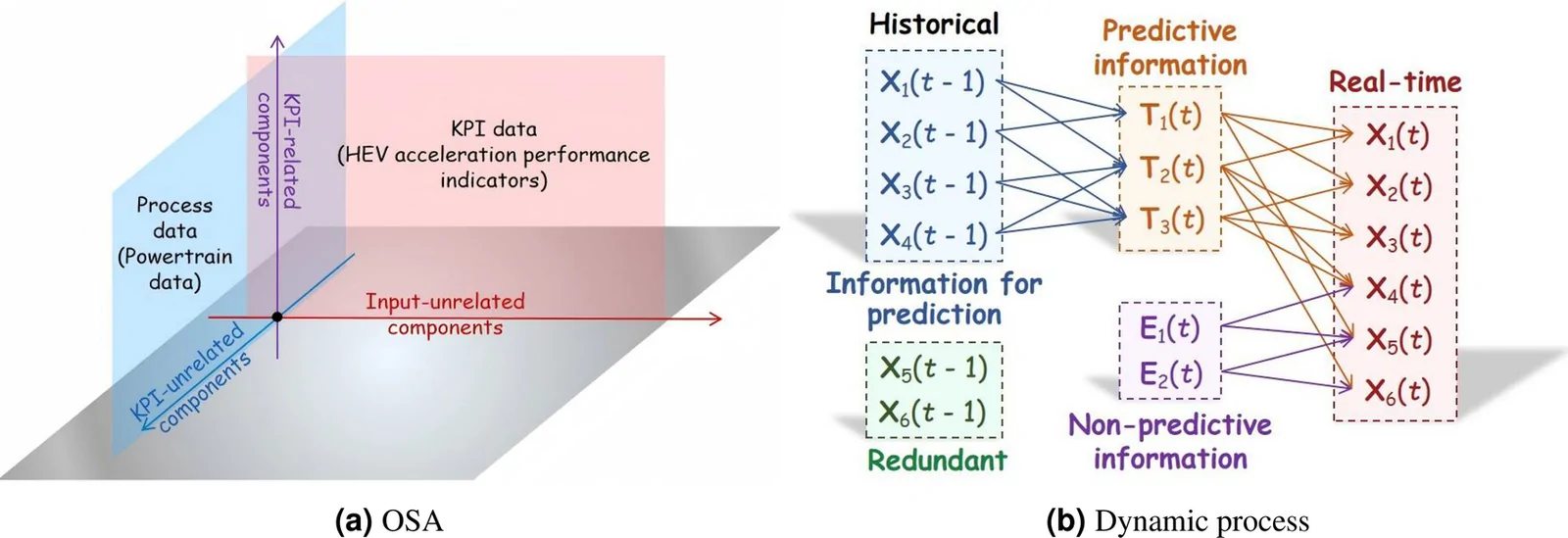
Principle of OSA and its dynamic problems.

OSA was originally proposed to extract common components shared by process variables ${{\bf X}}\in \mathbb {R}^{n\times m}$ and output variables ${{\bf Y}}\in \mathbb {R}^{n\times r}$. The common components are computed as

2 $$\begin{aligned} \hat{{{\bf X}}}_{\text {OSA}} = {{\bf Y}}({{\bf Y}}^{\text {T}} {{\bf Y}})^{-1} {{\bf Y}}^{\text {T}} {{\bf X}} = {{\bf T}}_{\text {com}}\Xi _{{{\bf X}}}^{\text {T}},\quad \hat{{{\bf Y}}}_{\text {OSA}} = {{\bf X}}({{\bf X}}^{\text {T}} {{\bf X}})^{-1} {{\bf X}}^{\text {T}} {{\bf Y}} = {{\bf T}}_{\text {com}}\Xi _{{{\bf Y}}}^{\text {T}}, \end{aligned}$$

where *p* is the number of PCs, $\Xi _{{{\bf X}}}\in \mathbb {R}^{m\times p}$ ($\Xi _{{{\bf X}}}^{\text {T}}\Xi _{{{\bf X}}} = {{\bf I}}$) and $\Xi _{{{\bf Y}}}\in \mathbb {R}^{r\times p}$ ($\Xi _{{{\bf Y}}}^{\text {T}}\Xi _{{{\bf Y}}} = {{\bf I}}$) are the transformation matrices. As shown above, both $\hat{{{\bf X}}}_{\text {OSA}}$ and $\hat{{{\bf Y}}}_{\text {OSA}}$ can be presented by linear combinations of the common latent variable ${{\bf T}}_{\text {com}}\in \mathbb {R}^{n\times p}$. Then OSA decomposes ${{\bf X}}$ and ${{\bf Y}}$ into bilinear terms:

3 $$\begin{aligned} {{\bf X}} = \hat{{{\bf X}}}_{\text {OSA}} + {{\bf E}}_{\text {OSA}},\quad {{\bf Y}} = \hat{{{\bf Y}}}_{\text {OSA}} + {{\bf F}}_{\text {OSA}}, \end{aligned}$$

where ${{\bf E}}_{\text {OSA}}$ and ${{\bf F}}_{\text {OSA}}$ are the residual matrices that do not contain information of ${{\bf T}}_{\text {com}}$. As shown in Fig. 2a , ${{\bf X}}$ and ${{\bf Y}}$ can be divided into three orthonormal subspaces: Common component subspace, i.e., $\hat{{{\bf X}}}_{\text {OSA}}$ and $\hat{{{\bf Y}}}_{\text {OSA}}$; Residual subspace of ${{\bf X}}$, that is, ${{\bf E}}_{\text {OSA}}$; Residual subspace of ${{\bf Y}}$, that is, ${{\bf F}}_{\text {OSA}}$.

### Problem statement

Based on the analysis of TLSM-based dynamic approaches and OSA methods, we identify three fundamental challenges in monitoring HEV powertrains:

1. The HEV powertrain exhibits significant dynamic characteristics. As shown in Eq. (1), the TLSM-based method addresses this issue by augmenting the data matrix with time-lagged observations, but this introduces redundant information that interferes with feature extraction.
2. The complex coupling among mechanical, electrical, and thermal subsystems introduces significant nonlinear behavior in HEV powertrains. Existing methods fail to simultaneously address the issues of dynamics, redundant information elimination, and nonlinearity.
3. In practical HEV operation, faults can occur with varying magnitudes. Existing methods lack theoretical guarantees on detection capability across different fault severities.

Given the above challenges, the core research question addressed in this paper is: *How can we simultaneously handle nonlinear dynamics while eliminating redundant information and ensuring consistent fault detection sensitivity across varying fault severities in HEV powertrain monitoring?* “Kernel dynamic OSA” of this paper is dedicated to addressing the above-mentioned issues.

## Kernel dynamic OSA

### Data structure definition

In this section, we first define the data structure used throughout the proposed method. Let ${{\bf X}} \in \mathbb {R}^{n \times m}$ denote the process data matrix, where *n* is the number of samples and *m* is the number of process variables. For dynamic process monitoring, we construct the real-time data matrix and historical data matrix as follows.

The real-time data matrix is defined as $${{\bf X}}(0) = \begin{bmatrix} {{\bf x}}_1(0)^{\text {T}}&{{\bf x}}_2(0)^{\text {T}}&\cdots&{{\bf x}}_n(0)^{\text {T}} \end{bmatrix}^{\text {T}} \in \mathbb {R}^{n \times m}$$, where ${{\bf x}}_i(0) \in \mathbb {R}^{m}$ represents the *i*th sample at the current time instant.

The historical data matrix is constructed by concatenating time-lagged observations as $\tilde{{{\bf Z}}} = \left[ {{\bf X}}(-1), {{\bf X}}(-2), \ldots , {{\bf X}}(-q) \right] \in \mathbb {R}^{n \times mq}$, where ${{\bf X}}(-j) \in \mathbb {R}^{n \times m}$ denotes the data matrix shifted *j* time steps into the past, and *q* is the maximum time lag order. Each row of $\tilde{{{\bf Z}}}$ can be expressed as $\tilde{{{\bf z}}}_i = \left[ {{\bf x}}_i(-1)^{\text {T}}, {{\bf x}}_i(-2)^{\text {T}}, \ldots , {{\bf x}}_i(-q)^{\text {T}} \right] ^{\text {T}} \in \mathbb {R}^{mq}$.

The core idea of the proposed KDOSA method is to apply OSA between the real-time data ${{\bf X}}(0)$ and the historical data $\tilde{{{\bf Z}}}$ in the kernel feature space, thereby separating the dynamic components from the static components while handling nonlinear relationships.

#### Remark 1

   In this paper, the terms “dynamic component” and “static component” refer specifically to the *predictable* and *unpredictable* portions of the real-time data ${{\bf X}}(0)$ with respect to the historical data $\tilde{{{\bf Z}}}$, respectively. This usage is distinct from the conventional meaning of “static process” in the control and signal processing literature, where “static” typically denotes a time-invariant process without temporal autocorrelation. In the present context, “static component” does not imply time-invariance; rather, it denotes the instantaneous component of ${{\bf X}}(0)$ that cannot be linearly predicted from $\tilde{{{\bf Z}}}$ in the kernel feature space. This distinction is maintained consistently throughout the manuscript, including in all figures and the online monitoring procedure.

### Drawbacks of TLSM-based dynamic approaches

While “Introduction” outlined the limitations of TLSM-based approaches qualitatively, this section provides a rigorous mathematical analysis to formally establish why these approaches suffer from the subspace division problem, a result that directly motivates the design of the proposed KDOSA method.

To understand why TLSM-based approaches suffer from the subspace division problem, we first analyze the information structure in dynamic processes which is shown in Fig. 2b . For a dynamic process, the real-time data include both information that can be predicted by historical data and information that cannot be predicted. The historical data also contain two types of information, that is, the information that can be used for prediction, and the information that cannot be used. For the above four types of information, the information that can be predicted by historical data is equal to the information that can be used for prediction, and the information that cannot be used for prediction does not belong to the real-time data.

To illustrate this problem mathematically, we study the situation that the maximum time lag *q = 1*, and the conclusion is applicable to other situations. Take ${{\bf S}}\in \mathbb {R}^{n\times p}$ as the independent components in process data ${{\bf X}}$, and the following:

4 $$\begin{aligned} {{\bf X}}(0) = {{\bf S}}(0){{\bf A}} + {{\bf S}}(-1){{\bf B}},\quad {{\bf X}}(-1) = {{\bf S}}(-1){{\bf A}} + {{\bf S}}(-2){{\bf B}}, \end{aligned}$$

where ${{\bf A}}$ and ${{\bf B}}$ are transformation matrices, ${{\bf S}}(-1){{\bf B}}$ (dynamic components) denotes information that can be predicted by historical data ${{\bf X}}(-1)$, ${{\bf S}}(0){{\bf A}}$ (static components) denotes information that cannot be predicted by ${{\bf X}}(-1)$, ${{\bf S}}(-1){{\bf A}}$ denotes information that can be used to predict real-time data ${{\bf X}}(0)$, and ${{\bf S}}(-2){{\bf B}}$ denotes information that cannot be used to predict ${{\bf X}}(0)$.

For TLSM-based dynamic approaches, the new data matrix is $\tilde{{{\bf X}}} = [{{\bf X}}(0), {{\bf X}}(-1)]$, and its covariance matrix is:

5 $$\begin{aligned} \tilde{{{\bf X}}}^{\text {T}}\tilde{{{\bf X}}} = \begin{bmatrix} {{\bf X}}(0)^{\text {T}} {{\bf X}}(0) & {{\bf X}}(-1)^{\text {T}} {{\bf X}}(0) \\ {{\bf X}}(0)^{\text {T}} {{\bf X}}(-1) & {{\bf X}}(-1)^{\text {T}} {{\bf X}}(-1) \end{bmatrix}. \end{aligned}$$

Taking $\text {tr}(\cdot )$ as the trace of one matrix, we get:

6 $$\begin{aligned} \begin{aligned} \text {tr}(\tilde{{{\bf X}}}^{\text {T}}\tilde{{{\bf X}}})&= \text {tr}({{\bf X}}(0)^{\text {T}} {{\bf X}}(0)) + \text {tr}({{\bf X}}(-1)^{\text {T}} {{\bf X}}(-1)) \\&= \text {tr}({{\bf A}}^{\text {T}} {{\bf S}}(0)^{\text {T}} {{\bf S}}(0){{\bf A}}) + \text {tr}({{\bf B}}^{\text {T}} {{\bf S}}(-2)^{\text {T}} {{\bf S}}(-2){{\bf B}}) \\&\quad + \text {tr}({{\bf B}}^{\text {T}} {{\bf S}}(-1)^{\text {T}} {{\bf S}}(-1){{\bf B}} + {{\bf A}}^{\text {T}} {{\bf S}}(-1)^{\text {T}} {{\bf S}}(-1){{\bf A}}). \end{aligned} \end{aligned}$$

When $n \rightarrow \infty$, one gets ${{\bf S}}(0)^{\text {T}} {{\bf S}}(0) = {{\bf S}}(-1)^{\text {T}} {{\bf S}}(-1) = {{\bf S}}(-2)^{\text {T}} {{\bf S}}(-2)$, and:

7 $$\begin{aligned} {\left\{ \begin{array}{ll} \text {tr}({{\bf B}}^{\text {T}} {{\bf S}}(-1)^{\text {T}} {{\bf S}}(-1){{\bf B}} + {{\bf A}}^{\text {T}} {{\bf S}}(-1)^{\text {T}} {{\bf S}}(-1){{\bf A}})> \text {tr}({{\bf A}}^{\text {T}} {{\bf S}}(0)^{\text {T}} {{\bf S}}(0){{\bf A}}) \\ \text {tr}({{\bf B}}^{\text {T}} {{\bf S}}(-1)^{\text {T}} {{\bf S}}(-1){{\bf B}} + {{\bf A}}^{\text {T}} {{\bf S}}(-1)^{\text {T}} {{\bf S}}(-1){{\bf A}}) > \text {tr}({{\bf B}}^{\text {T}} {{\bf S}}(-2)^{\text {T}} {{\bf S}}(-2){{\bf B}}) \end{array}\right. }, \end{aligned}$$

based on which we obtain that ${{\bf S}}(-1)$ related components have larger variance than the others and hence are more likely to be picked as PCs.

In addition, when $\text {tr}({{\bf B}}^{\text {T}} {{\bf S}}(-2)^{\text {T}} {{\bf S}}(-2){{\bf B}}) > \text {tr}({{\bf A}}^{\text {T}} {{\bf S}}(0)^{\text {T}} {{\bf S}}(0){{\bf A}})$, the ${{\bf S}}(-2)$ related components may be wrongly picked as PCs, which is useless for detecting fault occurrences in ${{\bf X}}(0)$, and ${{\bf S}}(0)$ related components may not be taken as PCs. In conclusion, traditional TLSM-based dynamic approaches may pay more attention to information that can be predicted by historical data and be less sensitive to faults in information that cannot be predicted by it. In addition, information not used for prediction may be wrongly taken as PCs, which reduces the sensitivity of the algorithm to real-time faults.

Based on the last section, one gets that when analysing the real-time and historical data in one space, then the dynamic and static components may be confused, which interfered with the monitoring performance. To avoid the abovementioned drawbacks, one solution is to analyse the real-time and historical data in separate subspaces, and hence the information in historical data will not be included in the PCs.

The components in real-time data ${{\bf X}}(0)$ that can be predicted by $\tilde{{{\bf Z}}}$ can be calculated as

8 $$\begin{aligned} \hat{{{\bf X}}}_{\text {OSA}} = \tilde{{{\bf Z}}} (\tilde{{{\bf Z}}}^{\text {T}}\tilde{{{\bf Z}}})^{-1}\tilde{{{\bf Z}}}^{\text {T}} {{\bf X}}(0), \end{aligned}$$

and those that cannot be predicted by $\tilde{{{\bf Z}}}$ are ${{\bf E}}_{\text {OSA}}={{\bf X}}(0)-\hat{{{\bf X}}}_{\text {OSA}}.$

The PCs in $\hat{{{\bf X}}}_{\text {OSA}}$ (dynamic component) can be obtained by PCA decomposition:

9 $$\begin{aligned} \hat{{{\bf X}}}_{\text {OSA}} = {{\bf T}}_{\text {dyn}} {{\bf P}}_{\text {dyn}}^{\text {T}} + {{\bf E}}_{\text {dyn}},\quad {{\bf T}}_{\text {dyn}} = \hat{{{\bf X}}}_{\text {OSA}} {{\bf P}}_{\text {dyn}}, \end{aligned}$$

where ${{\bf T}}_{\text {dyn}}\in \mathbb {R}^{n\times p}$ is the score matrix, ${{\bf P}}_{\text {dyn}}\in \mathbb {R}^{m\times p}$ is the loading matrix, and ${{\bf E}}_{\text {dyn}}$ is the residual matrix. In this step, the PCs are picked using the cumulative percent variance (CPV) method^31^, and the threshold follows the PCA criterion. In the decomposition, ${{\bf T}}_{\text {dyn}}$ is the dynamic component orthogonal to the static component ${{\bf E}}_{\text {OSA}}$.

As OSA only analyzes the components contained in ${{\bf X}}(0)$, the historical information does not interfere with the monitoring results, and hence it is more sensitive to process faults than TLSM-based methods.

### Proposed kernel dynamic OSA

Although the OSA-based dynamic method can effectively eliminate redundant information, it assumes linear relationships among variables, which limits its effectiveness in monitoring HEV powertrain systems that exhibit significant nonlinear behavior. To address this limitation, kernel methods are introduced to handle nonlinear relationships. Kernel methods map the original data into a high-dimensional feature space where nonlinear relationships become linear. Let $\phi (\cdot )$ denote the nonlinear mapping function: ${{\bf x}}_i \in \mathbb {R}^{m} \rightarrow \phi ({{\bf x}}_i) \in \mathcal {F} \subseteq \mathbb {R}^{N_\mathcal {F}}$, where $N_\mathcal {F}$ represents the dimensionality of the feature space which can be infinite for certain kernels.

In this paper, we employ the Gaussian kernel:

10 $$\begin{aligned} k({{\bf x}}_i, {{\bf x}}_j) = \phi ({{\bf x}}_i)^{\text {T}} \phi ({{\bf x}}_j) = \exp \left( -\frac{\Vert {{\bf x}}_i - {{\bf x}}_j\Vert ^2}{2\sigma ^2}\right) , \end{aligned}$$

where *σ* is the kernel width parameter that controls the smoothness of the mapping.

The core idea of the proposed KDOSA is to apply OSA between real-time process data ${{\bf X}}(0)$ and historical data $\tilde{{{\bf Z}}}$ in the kernel feature space, separating the dynamic components (predictable from history) from the static components (unpredictable). We first map both ${{\bf X}}(0)$ and $\tilde{{{\bf Z}}}$ into the kernel feature space using the Gaussian kernel. Let $\boldsymbol{\Phi }$ denotes the mapped data matrix in the feature space. Define the kernel matrices:

11 $$\begin{aligned} {\left\{ \begin{array}{ll} {{\bf K}}_{{{\bf X}}(0)} = \boldsymbol{\Phi }({{\bf X}}(0)) \boldsymbol{\Phi }({{\bf X}}(0))^{\text {T}}, [{{\bf K}}_{{{\bf X}}(0)}]_{ij} = k({{\bf x}}_i(0), {{\bf x}}_j(0)) \\ {{\bf K}}_{\tilde{{{\bf Z}}}} = \boldsymbol{\Phi }(\tilde{{{\bf Z}}}) \boldsymbol{\Phi }(\tilde{{{\bf Z}}})^{\text {T}}, [{{\bf K}}_{\tilde{{{\bf Z}}}}]_{ij} = k(\tilde{{{\bf z}}}_i, \tilde{{{\bf z}}}_j) \end{array}\right. }. \end{aligned}$$

The kernel matrices are centered as

12 $$\begin{aligned} \bar{{{\bf K}}}_{{{\bf X}}(0)} = {{\bf H}} {{\bf K}}_{{{\bf X}}(0)} {{\bf H}},\quad \bar{{{\bf K}}}_{\tilde{{{\bf Z}}}} = {{\bf H}} {{\bf K}}_{\tilde{{{\bf Z}}}} {{\bf H}}, \end{aligned}$$

where ${{\bf H}} = {{\bf I}}_n - \frac{1}{n} {{\bf 1}}_n{{\bf 1}}_n^{\text {T}}$ is the centering matrix and ${{\bf 1}}_n$ is an *n*-dimensional column vector of ones.

Following the OSA principle, the dynamic component (the part of ${{\bf X}}(0)$ that can be predicted by $\tilde{{{\bf Z}}}$) in the kernel feature space is ${{\bf K}}_{\text {dyn}} = \bar{{{\bf K}}}_{\tilde{{{\bf Z}}}} \left( \bar{{{\bf K}}}_{\tilde{{{\bf Z}}}} + \epsilon {{\bf I}}_n \right) ^{-1} \bar{{{\bf K}}}_{{{\bf X}}(0)}$, where *ε > 0* is a regularization parameter to ensure numerical stability. Then the static component (the part of ${{\bf X}}(0)$ that cannot be predicted by $\tilde{{{\bf Z}}}$) is ${{\bf K}}_{\text {sta}} = \bar{{{\bf K}}}_{{{\bf X}}(0)} - {{\bf K}}_{\text {dyn}}$.

#### Remark 2

 The decomposition ensures that ${{\bf K}}_{\text {dyn}}$ and ${{\bf K}}_{\text {sta}}$ are orthogonal in the kernel feature space. This orthogonality guarantees that there is no information leakage between the dynamic and static subspaces, enabling clean separation and independent monitoring. Unlike TLSM-based kernel methods such as DKPCA, the proposed KDOSA only analyzes the components contained in ${{\bf X}}(0)$, so the historical information does not interfere with the monitoring results.

To extract the PCs from both subspaces for monitoring purposes, we perform kernel PCA on the dynamic and static components separately.

For the dynamic component ${{\bf K}}_{\text {dyn}}$, we solve the eigenvalue problem: ${{\bf K}}_{\text {dyn}} \boldsymbol{\alpha }_i = \lambda _{\text {dyn},i} \boldsymbol{\alpha }_i$, where $\lambda _{\text {dyn},1} \ge \lambda _{\text {dyn},2} \ge \cdots \ge \lambda _{\text {dyn},n}$ are the eigenvalues and $\boldsymbol{\alpha }_1, \boldsymbol{\alpha }_2, \ldots , \boldsymbol{\alpha }_n$ are the corresponding eigenvectors. Select the first $d_{\text {dyn}}$ PCs based on the cumulative percent variance (CPV) criterion:

13 $$\begin{aligned} \text {CPV}(d) = \frac{\sum _{i=1}^{d} \lambda _i}{\sum _{i=1}^{n} \lambda _i}, \end{aligned}$$

where the CPV threshold is set to 85%, following the widely adopted convention in MSPM literature^32,33^. This value retains sufficient process variation for fault detection while excluding measurement noise in the residual subspace.

The score matrix for the dynamic subspace is ${{\bf T}}_{\text {dyn}} = {{\bf K}}_{\text {dyn}} {{\bf A}}_{\text {dyn}}$, where ${{\bf A}}_{\text {dyn}} = [\boldsymbol{\alpha }_1, \boldsymbol{\alpha }_2, \ldots , \boldsymbol{\alpha }_{d_{\text {dyn}}}] \in \mathbb {R}^{n \times d_{\text {dyn}}}$ with eigenvectors normalized such that $\boldsymbol{\alpha }_i^{\text {T}} \boldsymbol{\alpha }_i = 1/\lambda _{\text {dyn},i}$. Similarly, for the static component ${{\bf K}}_{\text {sta}}$, we solve the eigenvalue problem: ${{\bf K}}_{\text {sta}} \boldsymbol{\beta }_i = \lambda _{\text {sta},i} \boldsymbol{\beta }_i$, and select the first $d_{\text {sta}}$ principal components based on the CPV criterion. The score matrix for the static subspace is ${{\bf T}}_{\text {sta}} = {{\bf K}}_{\text {sta}} {{\bf A}}_{\text {sta}}$, where ${{\bf A}}_{\text {sta}} = [\boldsymbol{\beta }_1, \boldsymbol{\beta }_2, \ldots , \boldsymbol{\beta }_{d_{\text {sta}}}] \in \mathbb {R}^{n \times d_{\text {sta}}}$.

#### Remark 3

   The score matrices ${{\bf T}}_{\text {dyn}}$ and ${{\bf T}}_{\text {sta}}$ represent the dynamic and static information in the real-time data, respectively. Importantly, these score matrices do not contain the redundant historical information that plagues TLSM-based methods. The dynamic score matrix ${{\bf T}}_{\text {dyn}}$ captures only the predictable component of ${{\bf X}}(0)$ without including irrelevant historical noise, while the static score matrix ${{\bf T}}_{\text {sta}}$ captures the instantaneous variations that are independent of process history.

#### Remark 4

*Parameter selection:* For the unknown kernel parameter in the Gaussian function, the method commonly used in^34^ is applied to determine the kernel parameter in this article. First, a suitable false alarm rate (FAR) is selected for the normal training data. Then, the kernel parameter is iteratively adjusted until the FAR of the proposed method falls below a predetermined threshold. Moreover, the number of lags *q* is selected to be the lag minimizing the small sample Akaike information criterion (AIC)^35^.

### Process monitoring

#### Sliding window based $T^2$ indices

The $T^2$ index measures variations within the PC subspace. For the dynamic subspace, the $T^2$ index is defined as:

14 $$\begin{aligned} T^2_{\text {dyn}}(t) = \sum _{i=1}^{d_{\text {dyn}}} \frac{t_{\text {dyn},i}^2(t)}{\lambda _{\text {dyn},i}}, \end{aligned}$$

where $t_{\text {dyn},i}(t)$ is the *i*-th element of the dynamic score vector at time *t*, and $\lambda _{\text {dyn},i}$ is the corresponding eigenvalue. Similarly, for the static subspace:

15 $$\begin{aligned} T^2_{\text {sta}}(t) = \sum _{i=1}^{d_{\text {sta}}} \frac{t_{\text {sta},i}^2(t)}{\lambda _{\text {sta},i}}. \end{aligned}$$

To enhance monitoring robustness and reduce sensitivity to instantaneous noise, a sliding window of length *w* is applied to smooth the $T^2$ indices:

16 $$\begin{aligned} {\left\{ \begin{array}{ll} \bar{T}^2_{\text {dyn}}(t) = \frac{1}{w} \sum _{i=t-w+1}^{t} T^2_{\text {dyn}}(i) \\ \bar{T}^2_{\text {sta}}(t) = \frac{1}{w} \sum _{i=t-w+1}^{t} T^2_{\text {sta}}(i) \end{array}\right. }, \end{aligned}$$

where *t* is the current time index. The sliding window mechanism accumulates information over a sequence of samples, thereby improving fault detection reliability. The control limits $T^2_{\text {dyn,lim}}$ and $T^2_{\text {sta,lim}}$ are determined using kernel density estimation (KDE)^36^ with a confidence level of *1-γ* based on cross-validation.

The orthogonal subspace structure of KDOSA enables effective fault isolation through the following decision logic:

- Dynamic fault: If $\bar{T}^2_{\text {dyn}} > T^2_{\text {dyn,lim}}$, a dynamic fault is detected, indicating anomalies in the temporal dependencies of the process.
- Static fault: If $\bar{T}^2_{\text {sta}} > T^2_{\text {sta,lim}}$, a static fault is detected, indicating instantaneous deviations that are independent of process history.

This fault isolation capability provides valuable diagnostic information for maintenance decision-making, as operators can distinguish between faults that affect the temporal dynamics and those that cause instantaneous deviations.

For the sliding window width *w*, the randomized algorithm (RA) proposed in^37^ is applied to determine the appropriate window width in this article. Specifically, the expected fault detection rate (FDR) and FAR are first specified as target thresholds. The window width is then iteratively increased until the monitoring results satisfy both the expected FDR and FAR simultaneously, ensuring that the selected window width achieves the desired balance between detection sensitivity and false alarm suppression.

#### Online monitoring procedure

For a new test sample ${{\bf x}}_{\text {new}}(0)$ with its corresponding historical data $\tilde{{{\bf z}}}_{\text {new}}$, the online monitoring procedure is as follows:

Step 1: Compute the kernel vectors between the new sample and the training samples:

17 $$\begin{aligned} {\left\{ \begin{array}{ll} {{\bf k}}_{{{\bf X}}(0),\text {new}} = [k({{\bf x}}_{\text {new}}(0), {{\bf x}}_1(0)), \ldots , k({{\bf x}}_{\text {new}}(0), {{\bf x}}_n(0))]^{\text {T}} \\ {{\bf k}}_{\tilde{{{\bf Z}}},\text {new}} = [k(\tilde{{{\bf z}}}_{\text {new}}, \tilde{{{\bf z}}}_1), \ldots , k(\tilde{{{\bf z}}}_{\text {new}}, \tilde{{{\bf z}}}_n)]^{\text {T}} \end{array}\right. }. \end{aligned}$$

Step 2: Center the kernel vectors:

18 $$\begin{aligned} {\left\{ \begin{array}{ll} \bar{{{\bf k}}}_{{{\bf X}}(0),\text {new}} = {{\bf k}}_{{{\bf X}}(0),\text {new}} - \frac{1}{n} {{\bf K}}_{{{\bf X}}(0)} {{\bf 1}}_n - \frac{1}{n} {{\bf 1}}_n^{\text {T}} {{\bf k}}_{{{\bf X}}(0),\text {new}} {{\bf 1}}_n + \frac{1}{n^2} {{\bf 1}}_n^{\text {T}} {{\bf K}}_{{{\bf X}}(0)} {{\bf 1}}_n{{\bf 1}}_n \\ \bar{{{\bf k}}}_{\tilde{{{\bf Z}}},\text {new}} = {{\bf k}}_{\tilde{{{\bf Z}}},\text {new}} - \frac{1}{n} {{\bf K}}_{\tilde{{{\bf Z}}}} {{\bf 1}}_n - \frac{1}{n} {{\bf 1}}_n^{\text {T}} {{\bf k}}_{\tilde{{{\bf Z}}},\text {new}} {{\bf 1}}_n + \frac{1}{n^2} {{\bf 1}}_n^{\text {T}} {{\bf K}}_{\tilde{{{\bf Z}}}} {{\bf 1}}_n{{\bf 1}}_n \end{array}\right. }. \end{aligned}$$

Step 3: Compute the dynamic and static kernel components for the new sample:

19 $$\begin{aligned} {{\bf k}}_{\text {dyn,new}} = \bar{{{\bf k}}}_{\tilde{{{\bf Z}}},\text {new}}^{\text {T}} \left( \bar{{{\bf K}}}_{\tilde{{{\bf Z}}}} + \varepsilon {{\bf I}}_n \right) ^{-1} \bar{{{\bf K}}}_{{{\bf X}}(0)},\quad {{\bf k}}_{\text {sta,new}} = \bar{{{\bf k}}}_{{{\bf X}}(0),\text {new}}^{\text {T}} - {{\bf k}}_{\text {dyn,new}}. \end{aligned}$$

Step 4: Compute the score vectors:

20 $$\begin{aligned} {{\bf t}}_{\text {dyn,new}} = {{\bf k}}_{\text {dyn,new}} {{\bf A}}_{\text {dyn}},\quad {{\bf t}}_{\text {sta,new}} = {{\bf k}}_{\text {sta,new}} {{\bf A}}_{\text {sta}}. \end{aligned}$$

Step 5: Compute the monitoring indices $T^2_{\text {dyn}}$ and $T^2_{\text {sta}}$ using the score vectors.

Step 6: Apply sliding window smoothing to obtain $\bar{T}^2_{\text {dyn}}$ and $\bar{T}^2_{\text {sta}}$, then compare with their respective control limits. If $\bar{T}^2_{\text {dyn}} > T^2_{\text {dyn,lim}}$, a dynamic fault is detected; if $\bar{T}^2_{\text {sta}} > T^2_{\text {sta,lim}}$, a static fault is detected. In other words, if either of the two indicators exceeds its threshold, it indicates that a fault has occurred.

### Theoretical analysis of fault severity sensitivity

In this section, we provide a rigorous theoretical analysis of the proposed KDOSA method’s sensitivity to varying fault severities.

Let ${{\bf x}}(t) \in \mathbb {R}^m$ denote the process variable vector under normal operating conditions at time *t*. A fault occurring at time $t_0$ with a specific direction vector ${{\bf f}} \in \mathbb {R}^m$ and severity coefficient $\alpha \in \mathbb {R}^+$ can be modeled as ${{\bf x}}_f(t) = {{\bf x}}(t) + \alpha \cdot {{\bf f}}, \quad \forall t \ge t_0$, where ${{\bf x}}_f(t)$ denotes the fault-affected process variable vector and *α* represents the fault magnitude.

Then the kernel matrix element between ${{\bf x}}_f$ and a training sample ${{\bf x}}_i$ becomes

21 $$\begin{aligned} k({{\bf x}}_f, {{\bf x}}_i) = \exp \left( -\frac{\Vert {{\bf x}} + \alpha {{\bf f}} - {{\bf x}}_i\Vert ^2}{2\sigma ^2}\right) . \end{aligned}$$

This indicates that the fault signal $\alpha {{\bf f}}$ is non-linearly transformed. The sensitivity of the kernel value to *α* depends on the kernel width *σ* and the distance between the fault direction and the training data manifold. We now derive the minimum detectable fault severity for each subspace.

#### Theorem 3.1

*For a fault with severity* *α* *and direction* ${{\bf f}}$, *the fault is detectable in the dynamic subspace if*

22 $$\begin{aligned} \alpha > \alpha _{\min }^\text {dyn} = \frac{\sigma \cdot \sqrt{2\ln \left( \frac{T^2_\text {dyn, lim}}{\bar{T}^2_\text {dyn, normal}}\right) }}{\Vert {{\bf P}}_\text {dyn}\Phi ({{\bf f}})\Vert _{\mathcal {H}}}, \end{aligned}$$

*where* $\bar{T}^2_{\text {dyn, normal}}$ *denotes* $\bar{T}^2_{\text {dyn}}$ *indices under normal conditions; and* ${{\bf P}}_{\text {dyn}}$ *denotes the projection matrix for the dynamic subspace.*

*For a fault with severity* *α* *and direction* ${{\bf f}}$, *the fault is detectable in the static subspace if*

23 $$\begin{aligned} \alpha > \alpha _{\min }^\text {sta} = \frac{\sigma \cdot \sqrt{2\ln \left( \frac{T^2_\text {sta, lim}}{\bar{T}^2_\text {sta, normal}}\right) }}{\Vert {{\bf P}}_\text {sta}\Phi ({{\bf f}})\Vert _{\mathcal {H}}}, \end{aligned}$$

*where* $\bar{T}^2_{\text {sta, normal}}$ *denotes* $\bar{T}^2_{\text {sta}}$ *indices under normal conditions and* ${{\bf P}}_{\text {sta}}$ *denotes the projection matrix for the static subspace*.

#### Proof

The proofs for both subspaces follow identical logic. Taking the dynamic subspace as an example:

When a fault occurs, the kernel matrix perturbs as ${{\bf K}}_f = {{\bf K}} + \Delta {{\bf K}}(\alpha )$. For small *α*, we perform a first-order Taylor expansion:

24 $$\begin{aligned} k({{\bf x}}+\alpha {{\bf f}}, {{\bf x}}_i) \approx k({{\bf x}}, {{\bf x}}_i) + \alpha \cdot \frac{\partial k}{\partial \alpha }\bigg |_{\alpha =0}. \end{aligned}$$

The perturbation in the score vector is approximately linear with *α*: $\Delta {{\bf t}}_\text {dyn} \approx \alpha \cdot {{\bf v}}_\text {dyn}$, where ${{\bf v}}_\text {dyn} = {{\bf P}}_{dyn}\nabla _{\alpha }\phi ({{\bf x}})|_{\alpha =0}$. The change in the $T^2$ index is:

25 $$\begin{aligned} \Delta T^2_\text {dyn} \approx 2\alpha {{\bf t}}_\text {dyn, normal}^\text {T} \Lambda _\text {dyn}^{-1} {{\bf v}}_\text {dyn} + \alpha ^2 {{\bf v}}_\text {dyn}^\text {T} \Lambda _\text {dyn}^{-1} {{\bf v}}_\text {dyn}, \end{aligned}$$

where ${\Lambda }_{\text {dyn}}$ denotes the diagonal eigenvalue matrix for the dynamic subspace; and ${{\bf v}}_{\text {dyn}}$ denotes the projected fault gradient in the dynamic subspace. A fault is detected when $T^2_\text {dyn, normal} + \Delta T^2_\text {dyn} > T^2_\text {dyn, lim}$. For incipient faults, the linear term dominates. Solving for *α* and relating ${{\bf v}}_\text {dyn}$ to the projected fault norm $\Vert {{\bf P}}_\text {dyn}\phi ({{\bf f}})\Vert _{\mathcal {H}}$ yields Eq. (22).

The proof for the static subspace follows the same steps with ${{\bf P}}_\text {sta}$ and $\Lambda _\text {sta}$ replacing ${{\bf P}}_\text {dyn}$ and $\Lambda _\text {dyn}$, resulting in Eq. (23), where ${\Lambda }_{\text {sta}}$ denotes the diagonal eigenvalue matrix for the static subspace.

Proof finished.

## Case studies

To thoroughly demonstrate the performance of the proposed KDOSA method, we conducted a comparative analysis of OSA^27^, dynamic OSA^38^, deep OSA^28^ and KOSA^39^. OSA orthogonally decomposes process variables and KPI variables, yet it is a linear static method. Dynamic OSA addresses the dynamic issues of OSA using the TLSM method, but it does not consider nonlinearity. Deep OSA and KOSA tackle the nonlinear problems of OSA through deep structures and kernel functions, respectively, yet they fail to account for dynamic issues. FDR and FAR, are used to evaluate the performance of methods, which are expressed as

26 $$\begin{aligned} \text {FDR} = \dfrac{\text {No. of effective alarms}}{\text {No. of abnormal samples}} \times 100\%,\quad \text {FAR} = \dfrac{\text {No. of false alarms}}{\text {No. of normal samples}} \times 100\%. \end{aligned}$$

Typically, we hope that the higher the FDR, the better at the same FAR, and the lower the FAR, the better at the same FDR. Therefore, this paper adopts the performance index PI=FDR-FAR to evaluate and compare the performance of the algorithms. The experimental parameters are configured as Table. 1 shown.

**Table 1:** Experimental parameter settings comparison.

Parameter	Num. sim.	HEV	Parameter	Num. sim.	HEV
Training samples (*n*)	1000	1000	Gaussian kernel width ($\sigma ^2$)	200	200
Testing samples ($n_{\text {test}}$)	1000	1000	Regularization param. (*\varepsilon*)	$10^{-6}$	$10^{-6}$
Fault injection point	Sample 101	Sample 201	CPV threshold	95%	95%
Time lag order (*q*)	2	1	Sliding window length (*w*)	100	10
Significance level (*γ*)	0.01	0.15	Layer number of deep OSA	2	2

### Numerical simulation

To validate the effectiveness of the proposed KDOSA method in distinguishing dynamic and static faults, a numerical simulation study is conducted. The simulation model is designed to incorporate both nonlinear characteristics and dynamic temporal dependencies, which are representative of complex industrial processes such as HEV powertrains.

The numerical simulation model consists of four independent source signals $s_1, s_2, s_3, s_4$ that are transformed through nonlinear functions to generate six process variables. The model incorporates dynamic characteristics where the current observations depend on historical data. All simulations are performed in MATLAB R2019b with a fixed random seed (rng(42)) to ensure reproducibility. The source signals are generated from a uniform distribution as $s_i \sim \mathcal {U}(0, 1), \quad i = 1, 2, 3, 4$.

The static component of the process variables is defined through the following nonlinear transformations:

27 $$\begin{aligned} {{\bf X}}_{\text {static}}(t) = \begin{bmatrix} {{\bf X}}_1(t) \\ {{\bf X}}_2(t) \\ {{\bf X}}_3(t) \\ {{\bf X}}_4(t) \\ {{\bf X}}_5(t) \\ {{\bf X}}_6(t) \end{bmatrix} = \begin{bmatrix} s_1 + 3s_2 + 4s_3 + 2s_4 \\ 3\exp (\sin (s_1)) + \exp (s_2) + 2\exp (s_3) + 5\exp (s_4) \\ 2\exp (s_1) + 5s_2^2 + 3\cos (s_2^3) + 7s_4 \\ 2s_1^2 + s_2^3 + 3\sin (s_2^3) + \cos (s_4^2) \\ \sin (s_1^3) + \cos (s_2^3) + 3s_3 + s_4 \\ 5s_1^2 + 3\sin (s_2) + s_3 + 3\cos (s_4^2) \end{bmatrix} + {{\bf e}}_x(t), \end{aligned}$$

where ${{\bf e}}_x(t) \in \mathbb {R}^6$ represents measurement noise with $e_{x,i} \sim \mathcal {N}(0, 0.01^2)$.

The dynamic characteristics are introduced through a second-order autoregressive structure:

28 $$\begin{aligned} {{\bf X}}(t) = {{\bf X}}_{\text {static}}(t) + a_1 {{\bf X}}(t-1) + a_2 {{\bf X}}(t-2), \end{aligned}$$

where $a_1 = 0.3$ and $a_2 = 0.2$ are the dynamic coefficients that control the influence of historical observations on the current state. This formulation ensures that the process variables exhibit temporal autocorrelation, making the dataset suitable for evaluating dynamic monitoring methods.

In order to facilitate comparisons with other algorithms (such as KPI-related algorithms), this paper also establishes two KPI variables as

29 $$\begin{aligned} \begin{aligned} {{\bf Y}}&= \begin{bmatrix} 1 \cdot {{\bf X}}_1 + 0 \cdot {{\bf X}}_2 + 4 \sin \big ({{\bf X}}_3 {{\bf X}}_4(t)\big ) + 3 {{\bf X}}_5 + 1 \cdot {{\bf X}}_6 \\ 2 \cdot {{\bf X}}_1 + 0 \cdot {{\bf X}}_2 + 1 \cdot {{\bf X}}_4 + 3 \sin \big ({{\bf X}}_5\big ) + 0 \cdot {{\bf X}}_6 \end{bmatrix} + {{\bf e}}_y,\\ \end{aligned} \end{aligned}$$

where ${{\bf e}}_y \in \mathbb {R}^2$ represents measurement noise with $e_{y,i} \sim \mathcal {N}(0, 0.01^2)$.

To comprehensively evaluate the fault isolation capability of the proposed method, four types of faults are designed and the fault settings are summarized in Table 2.

**Table 2:** Fault settings for the numerical simulation.

Fault ID	Type	Description	Expected detection
Fault 1	Dynamic and static	$s_1\rightarrow {s}_1 + {{\bf 2.5}}$ (additive)	$T^2_{\text {dyn}}$ and $T^2_{\text {sta}}$
Fault 2	Dynamic and static	$s_2\rightarrow {s}_2 \times {{\bf 3.8}}$ (multiplicative)	$T^2_{\text {dyn}}$ and $T^2_{\text {sta}}$
Fault 3	Dynamic and static	$s_3\rightarrow {s}_3 + {{\bf 2.5}}$ (additive)	$T^2_{\text {dyn}}$ and $T^2_{\text {sta}}$
Fault 4	Dynamic and static	$s_4\rightarrow {s}_4 \times {{\bf 3.8}}$ (multiplicative)	$T^2_{\text {dyn}}$ and $T^2_{\text {sta}}$

These faults alter the instantaneous static relationships while preserving the underlying dynamic characteristics governed by coefficients $a_1$ and $a_2$. Hence, the static subspace index $T^2_{\text {sta}}$ serves as the primary detection tool. It is worth noting that, since the fault influence propagates over time, the dynamic subspace index $T^2_{\text {dyn}}$ should, in theory, also be capable of detecting such disturbances.

Table 3 presents the FDR and FAR for all methods over ten repeated trials. The proposed KDOSA method achieves outstanding performance: its dynamic subspace index $T^2_\text {dyn}$ yields FDRs of 98.3%, 98.0%, 98.3%, and 98.0% for Faults 1–4, respectively, while the static subspace index $T^2_\text {sta}$ reaches even higher values of 99.1%, 98.8%, 99.0%, and 98.9%. Both indices maintain zero FAR across all faults, demonstrating exceptional consistency and reliability. In contrast, baseline methods exhibit substantially lower detection capability and non-negligible false alarms: linear OSA attains at most 87.4% FDR (OSA-$T^2_E$ for Fault 4) and FARs up to 2.9%; Dynamic OSA peaks at 95.4% FDR (dynamic OSA-$T^2_E$ for Fault 2) but suffers from elevated FARs; Deep OSA and KOSA show intermediate performance yet still fall short of the proposed method.

**Table 3:** FDR and FAR comparison of the numerical simulation (%). Results with excellent performance are highlighted in bold, while the best-performing results are in italics.

Methods	Fault 1		Fault 2		Fault 3		Fault 4	
	FDR	FAR	FDR	FAR	FDR	FAR	FDR	FAR
OSA-$T^2_C$	41.3±2.2	0.4±0.7	71.5±2.7	1.1±1.1	38.6±2.9	1.5±1.5	7.0±1.8	0.9±1.1
OSA-$SPE_C$	47.2±1.5	1.5±1.5	**88.0**±**2.3**	2.9±2.0	25.7±2.4	2.7±1.8	**81.3**±**3.9**	2.3±1.9
OSA-$T^2_E$	62.5±2.1	1.0±1.1	**83.0**±**2.3**	1.4±1.5	53.7±3.2	1.0±0.8	**87.4**±**3.3**	1.8±1.6
OSA-$SPE_E$	6.1±0.9	1.1±0.7	**85.0**±**1.8**	1.7±1.9	6.1±1.2	1.1±1.2	64.3±3.7	1.4±1.4
OSA-$SPE_{XY}$	6.6±0.7	0.7±0.8	44.4±2.5	0.8±0.8	1.2±0.5	0.8±0.9	39.5±3.5	0.7±0.7
Dynamic OSA-$T^2_C$	71.9±2.7	0.8±0.9	**88.7**±**2.2**	2.4±2.3	**81.9**±**2.4**	2.2±3.1	24.0±3.0	1.7±2.3
Dynamic OSA-$SPE_C$	3.4±0.7	0.4±0.5	**85.3**±**1.8**	2.8±2.2	1.3±0.6	1.3±1.4	27.0±2.9	1.5±1.6
Dynamic OSA-$T^2_E$	**81.8**±**2.1**	1.4±2.0	**95.4**±**1.2**	4.7±4.5	69.3±3.2	1.4±1.6	**92.8**±**2.4**	3.2±3.9
Dynamic OSA-$SPE_E$	22.4±2.4	1.7±1.3	**91.0**±**2.0**	4.1±3.5	35.9±2.5	2.8±2.4	**83.7**±**3.8**	2.8±3.4
Dynamic OSA-$SPE_{XY}$	9.2±1.1	2.1±2.3	65.1±3.5	3.8±1.5	4.0±1.0	2.6±2.3	60.9±4.0	3.2±2.0
Deep OSA-$T^2_1$	41.3±2.2	0.4±0.7	71.5±2.7	1.1±1.1	38.6±2.9	1.5±1.5	7.0±1.8	0.9±1.1
Deep OSA-$SPE_1$	50.5±2.1	0.5±0.7	67.1±2.5	0.8±0.9	49.8±2.5	1.3±0.9	3.6±1.1	1.0±1.1
Deep OSA-$T^2_2$	64.3±2.1	1.3±0.7	61.0±2.8	1.2±1.2	**80.7**±**2.5**	1.6±1.5	12.5±2.2	1.1±1.3
Deep OSA-$SPE_2$	61.9±2.2	1.5±0.7	**82.1**±**2.3**	1.2±1.2	54.4±3.2	2.0±1.7	**87.8**±**3.3**	0.8±0.9
KOSA-$T^2_C$	55.2±2.5	0.7±0.9	74.3±3.0	1.1±1.2	60.3±2.8	1.4±1.6	61.1±4.1	0.9±1.1
KOSA-$SPE_C$	60.9±2.2	1.2±1.2	**84.3**±**2.5**	2.0±1.9	78.1±2.2	1.9±1.4	**87.7**±**2.9**	1.6±2.0
KOSA-$T^2_E$	58.1±2.3	0.9±1.0	**86.3**±**2.2**	1.6±1.8	71.5±2.7	1.6±1.3	**82.8**±**3.3**	1.6±1.8
KOSA-$SPE_E$	58.8±2.3	2.9±1.0	73.7±3.4	3.0±1.6	**81.0**±**2.3**	3.5±2.3	66.3±4.0	2.3±2.4
KOSA-$SPE_{XY}$	13.7±1.2	1.5±1.8	**85.3**±**1.5**	2.1±1.7	17.2±2.6	1.5±1.0	79.7±3.7	2.1±1.8
**Proposed-**$T^2_\text {dyn}$	***98.3***±***0.0***	***0.0***±***0.0***	***98.0***±***0.2***	***0.0***±***0.0***	***98.3***±***0.1***	***0.0***±***0.0***	***98.0***±***0.3***	***0.0***±***0.0***
**Proposed-**$T^2_\text {sta}$	***99.1***±***0.0***	***0.0***±***0.0***	***98.8***±***0.2***	***0.0***±***0.0***	***99.0***±***0.1***	***0.0***±***0.0***	***98.9***±***0.1***	***0.0***±***0.0***

Table 4 compares the fault detection speed of different methods. The metric fault detection delay (FDD) is defined as the number of samples between the occurrence of a fault and the first continuous alarm. The FDD values of the proposed method range from 9.0-19.4 samples across different fault scenarios, which is slightly higher compared to some baseline methods. This increase in detection delay is primarily attributed to the sliding window smoothing mechanism employed in the monitoring indices. In the early stages of a fault, the sliding window accumulates a significant amount of information from normal samples, rendering the algorithm less sensitive to incipient faults. Future work will focus on further improving this limitation.

**Table 4:** FDD comparison of the numerical simulation. Given that a critically low FDR indicates a failure to effectively detect faults, the FDD index is deemed invalid and is not reported.

Methods	Fault 1	Fault 2	Fault 3	Fault 4	Methods	Fault 1	Fault 2	Fault 3	Fault 4
OSA-$T^2_C$	4.8±3.7	2.9±3.0	6.6±3.0	36.8±38.8	Deep OSA-$SPE_1$	3.5±1.8	3.0±3.0	6.5±4.7	–
OSA-$SPE_C$	5.4±3.5	1.8±1.3	5.2±4.2	4.0±2.5	Deep OSA-$T^2_2$	4.3±4.1	3.7±3.2	3.1±1.9	16.0±8.4
OSA-$T^2_E$	4.1±3.4	3.6±3.1	2.6±2.5	3.5±1.4	Deep OSA-$SPE_2$	4.5±4.0	3.8±3.2	3.6±2.0	14.6±9.0
OSA-$SPE_E$	–	2.5±2.9	–	8.8±7.7	KOSA-$T^2_C$	5.9±4.8	3.2±3.0	2.9±2.0	6.6±5.8
OSA-$SPE_{XY}$	–	4.9±4.0	–	9.9±6.8	KOSA-$SPE_C$	4.4±2.5	2.4±2.4	1.9±1.1	3.1±1.5
Dynamic OSA-$T^2_C$	4.1±4.4	2.4±2.3	3.8±2.3	11.8±8.6	KOSA-$T^2_E$	4.5±4.0	2.6±2.3	2.6±1.3	4.6±3.2
Dynamic OSA-$SPE_C$	–	2.0±2.1	–	15.1±13.8	KOSA-$SPE_E$	4.5±4.0	2.6±2.3	2.6±1.3	4.6±3.2
Dynamic OSA-$T^2_E$	5.2±4.1	2.1±2.3	2.7±2.2	2.0±1.5	KOSA-$SPE_{XY}$	10.8±7.6	2.5±2.4	5.8±4.9	4.0±2.5
Dynamic OSA-$SPE_E$	12.2±10.7	2.1±2.3	4.4±3.8	2.5±2.2	Proposed-$T^2_\text {dyn}$	16.0±0.0	19.4±2.0	16.4±0.5	18.8±2.6
Dynamic OSA-$SPE_{XY}$	–	2.8±2.5	–	6.4±5.4	Proposed-$T^2_\text {sta}$	9.0±0.0	11.5±2.0	9.7±0.5	10.7±1.3
Deep OSA-$T^2_1$	4.8±3.7	2.9±3.0	6.6±3.0	–					

**Fig. 3:**
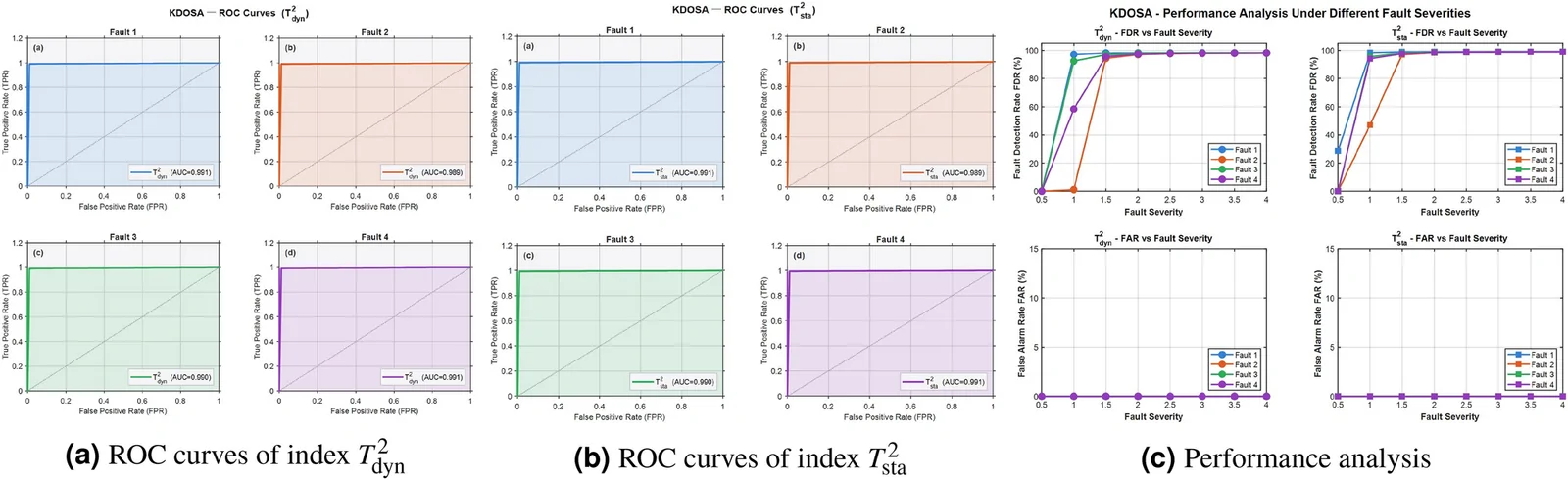
ROC curves and performance analysis under different fault severities.

Figure 3a, b present the ROC curves for the numerical simulation across four fault scenarios. The proposed KDOSA method demonstrates exceptional performance, with both $T^2_\text {dyn}$ and $T^2_\text {sta}$ monitoring indices achieving area under the curve (AUC) values consistently exceeding 0.994 across all cases. The ROC curves closely approximate the top-left corner of the plot, indicating that KDOSA maintains extremely high true positive rates (TPR) even at negligible false positive rates (FPR). This superior performance confirms the theoretical advantage of KDOSA in eliminating the redundant information problem inherent in traditional TLSM-based approaches. By orthogonally decomposing real-time data into predictable and unpredictable subspaces within the kernel feature space, KDOSA effectively captures complex nonlinear and dynamic characteristics without contamination from irrelevant historical data.

To validate the performance under varying fault severity levels, eight fault scenarios were configured with the fault magnitude coefficient ranging from 0.5 to 4.0. The mean values of the FDR and FAR are calculated over 10 repeated experiments, with the results presented in Fig. 3c . As shown, the FDR is 0% for all faults at low fault severity, indicating that incipient faults below a certain threshold cannot be detected. When the fault magnitude reaches 1.0, the FDR increases from 97.2% to 98.3%. Moreover, the FAR remained 0 across all severity levels, confirming the robustness of the KDOSA method. The experimental results validate the theoretical analysis in “Theoretical analysis of fault severity sensitivity”, showing that the proposed method achieves high detection sensitivity once the fault severity exceeds the minimum detectable threshold.

The FDR values of the proposed method under different fault severity levels are presented in Table 5. The FAR is not reported in the table, as it remained zero across all scenarios. As shown, the FDR saturates at approximately 98% when magnitude *≥ 2.0*, indicating that further increases in fault severity do not significantly improve detection performance.

**Table 5:** FDR performance under different fault severities. Results with the best-performing results are bold.

Magnitude	Fault 1	Fault 2	Fault 3	Fault 4	Magnitude	Fault 1	Fault 2	Fault 3	Fault 4
0.5	0.0±0.0	0.0±0.0	0.0±0.0	0.0±0.0	2.5	**98.3±0.0**	97.8±0.4	**98.3±0.1**	97.9±0.3
1.0	97.2±0.3	1.1±3.3	92.6±0.7	58.5±11.5	3.0	**98.3±0.0**	98.0±0.2	**98.3±0.0**	98.1±0.2
1.5	98.2±0.1	94.5±1.2	97.1±0.2	95.8±0.9	3.5	**98.3±0.0**	98.1±0.2	**98.3±0.0**	98.1±0.2
2.0	**98.3**±**0.0**	97.2±0.5	98.0±0.1	97.5±0.4	4.0	**98.3**±**0.0**	**98.2**±**0.2**	**98.3**±**0.0**	**98.2**±**0.1**

### HEV powertrain system

The proposed KDOSA and comparative algorithms are tested in a hybrid electric bus powertrain system manufactured by a certain brand and operated in Xiamen, China. The data are collected with a sampling period of 100 Hz. The technical specifications are shown in Table 6.

**Table 6:** Technical specifications.

Parameter	Value	Location	Parameter	Value	Location
Peak power	135kW	Motor	Maximum torque	850Nm	Motor
Rated power	85kW	Motor	Rated torque	500Nm	Motor
Maximum speed	3000r/min	Motor	Rated power	147kW	Engine
Rated speed	1700r/min	Motor	Rated torque	730Nm	Engine

Table 7 summarizes the 32 measured variables used for HEV powertrain fault detection in this study. These variables are collected from multiple subsystems including the engine, driving motor, ISG motor, battery system, cooling system, lubrication system, and fuel system. Among these variables, four are selected as KPI variables (**Y**): actual engine torque, driving motor speed, engine speed, and motor torque. These KPIs directly reflect the dynamic performance output of the hybrid powertrain system. The remaining 28 variables serve as process variables (**X**), which represent intermediate states such as temperatures, pressures, currents, and operating conditions that influence the overall system performance.

**Table 7:** Measured variables for HEV powertrain fault detection. **X**: process variables; **Y**: KPI variables.

No.	Variable	Unit	Type	No.	Variable	Unit	Type
1	Actual engine torque	Nm	**Y**	17	Engine oil temperature	$^{\circ }$C	**X**
2	Coolant level	*%*	**X**	18	EV Air pump temperature	$^{\circ }$C	**X**
3	Coolant pressure	kPa	**X**	19	Exhaust gas mass flow	kg/h	**X**
4	Crankcase pressure	kPa	**X**	20	Crankcase blow-by	L/min	**X**
5	Demand speed	r/min	**X**	21	Fuel delivery pressure	kPa	**X**
6	Demand torque	Nm	**X**	22	Fuel temperature	$^{\circ }$C	**X**
7	Driver demand engine	*%*	**X**	23	ISG inverter temp.	$^{\circ }$C	**X**
8	Driving motor speed	r/min	**Y**	24	ISG motor speed	r/min	**X**
9	Driving motor temperature	$^{\circ }$C	**X**	25	ISG motor temp.	$^{\circ }$C	**X**
10	Engine speed	r/min	**Y**	26	MCU temperature	$^{\circ }$C	**X**
11	Accelerator pedal	*%*	**X**	27	Motor AC current	A	**X**
12	Engine coolant temperature	$^{\circ }$C	**X**	28	Motor rotation count	−	**X**
13	Engine desired operation	*%*	**X**	29	Motor torque	Nm	**Y**
14	Engine oil level	*%*	**X**	30	Winding temperature	$^{\circ }$C	**X**
15	Engine oil pressure	kPa	**X**	31	Battery voltage	V	**X**
16	Engine operating speed	r/min	**X**	32	Battery current	A	**X**

The training data are collected during a real-world driving trip from Xiamen Railway Station to Xiamen University, covering typical urban bus operating conditions including frequent acceleration, deceleration, and idling events. This experiment collects a total of four fault types that represent typical powertrain performance degradation scenarios encountered in real-world HEV operations:

- Fault 1: insufficient vehicle power. This fault manifests as a significant reduction in the vehicle’s driving capability, where the powertrain fails to deliver the expected torque and power output despite normal driver inputs. The root cause typically involves degradation in the power transmission path, such as clutch slippage, torque converter inefficiency, or mechanical wear in the drivetrain components. This fault directly affects the dynamic performance of the vehicle and is reflected in the KPI variables including actual engine torque and motor torque.
- Fault 2: excessive battery cell voltage deviation. This fault occurs when individual cells within the battery pack exhibit abnormal voltage differences exceeding the acceptable threshold. Such imbalance indicates potential cell degradation, internal resistance variations, or thermal management issues. The excessive voltage deviation can lead to reduced battery capacity, compromised energy efficiency, and potential safety hazards. This fault primarily affects the battery voltage and current measurements and subsequently influences the overall powertrain performance.
- Fault 3: high-speed driving level-3 alarm. This represents a critical system-level fault that triggers during high-speed operation, typically indicating severe abnormalities in the powertrain control system. Level-3 alarms are the most serious category in the vehicle diagnostic hierarchy, often requiring immediate intervention. This fault may involve multiple subsystem malfunctions such as motor overheating, inverter failures, or communication errors between control units. The fault signature propagates through both dynamic and static process relationships.
- Fault 4: throttle response failure. This fault is characterized by the vehicle’s inability to respond to accelerator pedal inputs, representing a critical disconnection between the driver command and powertrain actuation. Potential causes include electronic throttle control malfunctions, accelerator pedal sensor failures, or communication faults in the CAN bus network. This fault severely impacts vehicle drivability and safety, as the powertrain cannot execute the demanded torque and speed profiles.

These four fault types cover a comprehensive range of powertrain anomalies, including mechanical degradation, electrochemical imbalance, system-level critical failures, and control signal interruption. The diversity of these faults provides a rigorous testbed for evaluating the proposed KDOSA method’s capability in detecting various types of performance-related anomalies in complex HEV systems.

Table 8 presents a comparative evaluation of five algorithms using the PI index, which effectively eliminates potential bias arising from varying control limits across different methods. Values exceeding 90% are highlighted in bold to indicate satisfactory detection performance, while the best-performing results are additionally given in italics.

#### Remark 4

    The proposed KDOSA-$T^2_\text {sta}$ statistic exhibits superior sensitivity to powertrain-related anomalies in HEVs. Consequently, this comparative study focuses exclusively on this statistic against the benchmark algorithms.

#### Remark 5

Given that all fault scenarios considered in this study are Y-related, the $T^2_E$ and $SPE_E$ statistics of OSA, dynamic OSA, and KOSA–which are specifically designed for detecting Y-unrelated faults–are excluded from the comparative analysis.

**Table 8:** FAR, FDR and PI comparison for HEV powertrain fault detection. The smaller the FAR value, the better; the larger the PI value, the better.

Methods	Fault 1			Fault 2			Fault 3			Fault 4		
	FAR	FDR	PI	FAR	FDR	PI	FAR	FDR	PI	FAR	FDR	PI
OSA-$T^2_C$	1.5%	36.3%	34.8%	1.5%	100.0%	**98.5%**	1.5%	100.0%	**98.5%**	1.5%	75.1%	73.6%
OSA-$SPE_C$	1.5%	75.5%	74.0%	1.5%	100.0%	**98.5%**	1.5%	100.0%	**98.5%**	1.5%	100.0%	**98.5%**
OSA-$SPE_{XY}$	3.0%	6.5%	3.5%	3.0%	100.0%	**97.0%**	3.0%	94.3%	**91.3%**	3.0%	8.8%	5.8%
Dynamic OSA-$T^2_C$	10.5%	29.0%	18.5%	10.5%	100.0%	89.5%	10.5%	100.0%	89.5%	10.5%	100.0%	89.5%
Dynamic OSA-$SPE_C$	***0.0%***	0.0%	0.0%	***0.0%***	0.0%	0.0%	***0.0%***	0.0%	0.0%	***0.0%***	0.0%	0.0%
Dynamic OSA-$SPE_{XY}$	40.5%	60.3%	19.8%	40.5%	100.0%	59.5%	40.5%	100.0%	59.5%	40.5%	100.0%	59.5%
Deep OSA-$T^2_1$	1.5%	36.3%	34.8%	1.5%	100.0%	**98.5%**	1.5%	100.0%	**98.5%**	1.5%	75.1%	73.6%
Deep OSA-$SPE_1$	1.5%	1.5%	0.0%	1.5%	46.9%	45.4%	1.5%	51.6%	50.1%	1.5%	1.5%	0.0%
Deep OSA-$T^2_2$	1.0%	1.1%	0.1%	1.0%	100.0%	**99.0%**	1.0%	98.8%	**97.8%**	1.0%	23.2%	22.2%
Deep OSA-$SPE_2$	0.5%	1.3%	0.8%	0.5%	70.3%	69.8%	0.5%	91.4%	**90.9%**	0.5%	21.1%	20.6%
KOSA-$T^2_C$	1.0%	67.9%	66.9%	1.0%	100.0%	99.0%	1.0%	100.0%	99.0%	1.0%	100.0%	99.0%
KOSA-$SPE_C$	0.5%	48.0%	47.5%	0.5%	100.0%	**99.5%**	0.5%	100.0%	**99.5%**	0.5%	100.0%	**99.5%**
KOSA-$SPE_{XY}$	0.5%	53.6%	53.1%	0.5%	100.0%	**99.5%**	0.5%	97.3%	**96.8%**	0.5%	100.0%	**99.5%**
**Proposed KDOSA-** $T^2_\text {sta}$	***0.0%***	99.9%	***99.9%***	***0.0%***	99.9%	***99.9%***	***0.0%***	99.9%	***99.9%***	***0.0%***	99.9%	***99.9%***

The proposed KDOSA method achieves exceptional performance across all four fault scenarios, attaining a PI of 99.9% for each fault type with zero false alarms. This consistency underscores the robustness of kernel-based orthogonal subspace decomposition in capturing complex nonlinear dynamics inherent to HEV powertrains. In contrast, baseline methods exhibit performance degradation that varies with fault characteristics. The linear static method OSA delivers satisfactory results for Faults 2, 3, and 4 using its $SPE_C$ index, but struggles with Fault 1, achieving only 34.8% PI for $T^2_C$ and 74.0% for $SPE_C$. This discrepancy suggests that Fault 1 involves nonlinear dynamics beyond the capacity of linear methods. Dynamic OSA, which incorporates temporal dependencies via a time-lagged strategy, does not consistently outperform static OSA in this application. Notably, its $SPE_C$ index yields 0.0% PI across all faults, indicating complete detection failure. This is attributed to the redundant information problem inherent in TLSM-based approaches, where irrelevant historical data contaminates feature extraction. The $T^2_C$ index of dynamic OSA also shows elevated FARs, highlighting the limitations of naive temporal augmentation without proper subspace separation. The kernel-based KOSA method improves over linear approaches, achieving PI above 99% for Faults 2, 3, and 4 across multiple statistics. However, it still underperforms on Fault 1, with $T^2_C$ reaching only 66.9% PI and $SPE_C$ 47.5%. This limitation arises because KOSA addresses nonlinearity but fails to separate dynamic and static components within the kernel feature space. Deep OSA exhibits inconsistent performance across faults and indices. While its $T^2_1$ index achieves 98.5% PI for Faults 2 and 3, it drops to 34.8% for Fault 1 and 73.6% for Fault 4. The *SPE* indices perform poorly overall, with $SPE_1$ yielding 0.0% PI for both Fault 1 and Fault 4. These results indicate that hierarchical decomposition alone is insufficient for addressing the complex dynamic nonlinear coupling in HEV powertrains.

Table 9 compares the FDD across different methods for the HEV powertrain experiments. As shown, the proposed KDOSA-$T^2_\text {sta}$ achieves FDD values of 2 samples across all four fault types, demonstrating consistent and reliable detection timing. It should be acknowledged that the sliding window smoothing mechanism employed in the KDOSA monitoring indices introduces a certain detection delay. During the initial stages of fault occurrence, the sliding window accumulates information from both normal and faulty samples, which reduces sensitivity to incipient faults. This represents a trade-off between detection robustness and response speed. While some comparative methods exhibit marginally lower FDD values in certain scenarios, they often suffer from substantially lower FDRs or elevated FARs.

**Table 9:** FDD comparison of the HEV powertrain. Given that a critically low FDR indicates a failure to effectively detect faults, the FDD index is deemed invalid and is not reported.

Methods	Fault 1	Fault 2	Fault 3	Fault 4	Methods	Fault 1	Fault 2	Fault 3	Fault 4
OSA-$T^2_C$	1	1	1	1	Deep OSA-$T^2_2$	–	1	1	47
OSA-$SPE_C$	1	1	1	1	Deep OSA-$SPE_2$	–	1	1	47
OSA-$SPE_{XY}$	1	1	1	–	KOSA-$T^2_C$	1	1	1	1
Dynamic OSA-$T^2_C$	1	1	1	1	KOSA-$SPE_C$	1	1	1	1
Dynamic OSA-$SPE_C$	–	–	–	–	KOSA-$SPE_E$	1	1	1	1
Dynamic OSA-$SPE_{XY}$	1	1	1	1	KOSA-$SPE_{XY}$	1	1	1	1
Deep OSA-$T^2_1$	1	1	1	1	Proposed-$T^2_{\text {sta}}$	2	2	2	2
Deep OSA-$SPE_1$	–	5	1	–					

**Fig. 4:**
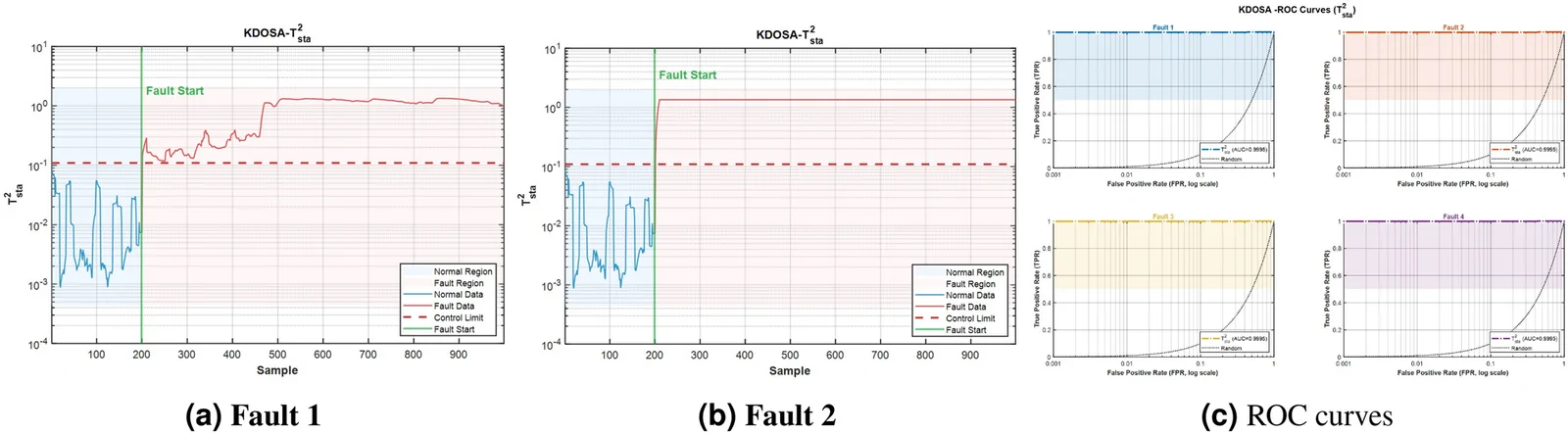
Monitoring results of the proposed KDOSA.

**Fig. 5:**
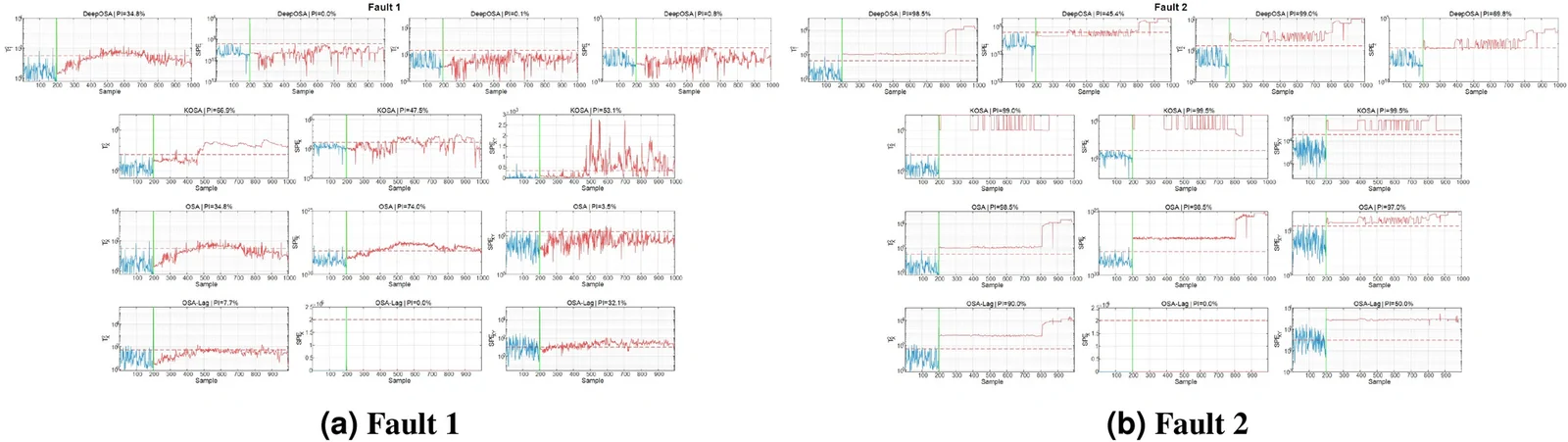
Monitoring charts of comparative algorithms for Fault 1 and Fault 2.

Taking Fault 1 and Fault 2 as examples, the fault detection plots of the proposed KDOSA method are shown in Fig. 4a, b, while those of the comparative algorithms are shown in Figs. 5(a) and 5(b).

Figure 4a, b present the monitoring charts of the proposed KDOSA method for Faults 1 and Fault 2. In both cases, the static subspace index $T^2_{\text {sta}}$ remains well below the control limit during the normal operating region, demonstrating the method’s ability to distinguish between normal process variations and genuine fault conditions. Upon fault injection at sample 201, the monitoring index responds promptly by exceeding the control limit, achieving rapid fault detection with minimal delay. Figure 4c illustrates the ROC curves of the proposed KDOSA-$T^2_\text {sta}$ index for the HEV powertrain fault detection across four fault types. As shown, all four fault scenarios achieve an identical AUC value of 0.9995, with the ROC curves nearly coinciding with the top-left boundary of the plot. This indicates that KDOSA maintains a TPR of 1.0 even when the FPR is as low as 0.001. The substantial separation between the KDOSA curves and the random classifier baseline (dotted line) further validates the method’s discriminative power. Notably, the consistent performance across Fault 1 through Fault 4 demonstrates KDOSA’s robustness in handling diverse fault mechanisms in complex HEV systems. The near-perfect AUC values confirm that the orthogonal decomposition in kernel space effectively isolates fault-related variations from normal operational dynamics.

Figure 5a, b illustrate the monitoring charts of comparative algorithms for Faults 1 and 2, respectively. For Fault 1, the OSA-$T^2_X$ chart shows limited separation between normal and faulty regions, while OSA-Lag-SPEC completely fails to respond to the fault. KOSA demonstrates better performance than linear methods but still exhibits overlap between normal and fault regions. In contrast, for Fault 2, most methods show clearer fault signatures, with OSA, KOSA, and deep OSA achieving PI values above 90% for at least one monitoring index. However, the KDOSA method consistently outperforms all alternatives with its superior separation between operating conditions.

## Conclusion

This paper proposed a KDOSA method for monitoring faults in HEV powertrain systems. The proposed method aims to address the nonlinear characteristics inherent in HEV powertrains and the redundant information problem in traditional TLSM-based dynamic approaches. First, we extended OSA to the kernel feature space using Gaussian kernel functions, with the goal of capturing complex nonlinear relationships among process variables. Second, we provided theoretical analysis suggesting that the proposed KDOSA method can alleviate the redundant information problem by orthogonally decomposing real-time data into dynamic and static components in the reproducing kernel Hilbert space. Third, we developed a comprehensive monitoring framework with $T^2$ indices for both dynamic and static subspaces, which can facilitate fault detection and provide diagnostic information about whether faults originate from temporal dependencies or instantaneous deviations.

The effectiveness of the proposed method was examined through both numerical simulations and real-world HEV powertrain experiments. In numerical simulations involving nonlinear dynamic processes with four fault types, KDOSA achieved PI values of 96.6–99.7% without triggering false alarms in the tested scenarios, showing improved performance over comparative methods. In the HEV powertrain case study, KDOSA achieved 99.9% PI with no false alarms observed in the tested cases across all fault types, suggesting the potential of KDOSA in handling complex nonlinear dynamic faults under the conditions evaluated.

The experimental results indicate that the combination of kernel-based nonlinear modeling and orthogonal dynamic-static separation can be beneficial for fault detection in HEV powertrain systems. Generalization of these findings to other operating conditions or HEV configurations would require further investigation. The limitation of this work lies in the fact that, for dynamic nonlinear processes, the sliding window mechanism exhibits reduced sensitivity to incipient faults. Specifically, the window-based computation introduces an inherent detection delay, as a sufficient number of new observations must accumulate before a fault can be reflected in the extracted features. This represents a trade-off between the robustness provided by the sliding-window approach and its responsiveness to slowly developing or early-stage faults. Future work could also extend such algorithms to enable the monitoring of non-Gaussian, non-stationary, and multi-mode processes.

## Data Availability

The datasets used and/or analysed during the current study are available from the corresponding author on reasonable request.
